# Safety and Feasibility of Intraoperative High PEEP Titrated to the Lowest Driving Pressure (ΔP)—Interim Analysis of DESIGNATION

**DOI:** 10.3390/jcm13010209

**Published:** 2023-12-29

**Authors:** Sunny G. L. H. Nijbroek, Liselotte Hol, Ary Serpa Neto, David M. P. van Meenen, Sabrine N. T. Hemmes, Markus W. Hollmann, Marcus J. Schultz

**Affiliations:** 1 Department of Anesthesiology, Amsterdam UMC Location AMC, 1105 AZ Amsterdam, The Netherlands; sunny.nijbroek@radboudumc.nl (S.G.L.H.N.); l.hol@amsterdamumc.nl (L.H.); d.m.vanmeenen@amsterdamumc.nl (D.M.P.v.M.); m.w.hollmann@amsterdamumc.nl (M.W.H.); 2 Department of Anesthesiology, Radboudumc, 6525 GA Nijmegen, The Netherlands; 3 Department of Intensive Care, Amsterdam UMC Location AMC, 1105 AZ Amsterdam, The Netherlands; ary.serpaneto@monash.edu; 4 Department of Critical Care Medicine, Australian and New Zealand Intensive Care Research Centre (ANZIC-RC), Monash University, Melbourne, VIC 3004, Australia; 5 Department of Anesthesiology, The Netherlands Cancer Institute—Antoni van Leeuwenhoek Hospital, 1066 CX Amsterdam, The Netherlands; s.hemmes@nki.nl; 6 Mahidol–Oxford Tropical Medicine Research Unit (MORU), Mahidol University, Bangkok 10400, Thailand; 7 Nuffield Department of Medicine, University of Oxford, Oxford OX3 7BN, UK

**Keywords:** anesthesia, intraoperative ventilation, ventilation, positive end–expiratory pressure, PEEP, recruitment maneuver, RM, driving pressure, safety, feasibility, interim analysis

## Abstract

Uncertainty remains about the best level of intraoperative positive end–expiratory pressure (PEEP). An ongoing RCT (‘DESIGNATION’) compares an ‘individualized high PEEP’ strategy (‘iPEEP’)—titrated to the lowest driving pressure (ΔP) with recruitment maneuvers (RM), with a ‘standard low PEEP’ strategy (‘low PEEP’)—using 5 cm H_2_O without RMs with respect to the incidence of postoperative pulmonary complications. This report is an interim analysis of safety and feasibility. From September 2018 to July 2022, we enrolled 743 patients. Data of 698 patients were available for this analysis. Hypotension occurred more often in ‘iPEEP’ vs. ‘low PEEP’ (54.7 vs. 44.1%; RR, 1.24 (95% CI 1.07 to 1.44); *p* < 0.01). Investigators were compliant with the study protocol 285/344 patients (82.8%) in ‘iPEEP’, and 345/354 patients (97.5%) in ‘low PEEP’ (*p* < 0.01). Most frequent protocol violation was missing the final RM at the end of anesthesia before extubation; PEEP titration was performed in 99.4 vs. 0%; PEEP was set correctly in 89.8 vs. 98.9%. Compared to ‘low PEEP’, the ‘iPEEP’ group was ventilated with higher PEEP (10.0 (8.0–12.0) vs. 5.0 (5.0–5.0) cm H_2_O; *p* < 0.01). Thus, in patients undergoing general anesthesia for open abdominal surgery, an individualized high PEEP ventilation strategy is associated with hypotension. The protocol is feasible and results in clear contrast in PEEP. DESIGNATION is expected to finish in late 2023.

## 1. Introduction

Intraoperative lung-protective ventilation has the potential to protect against postoperative pulmonary complications, and one important element may be to use an appropriate level of positive end–expiratory pressure (PEEP). Intraoperative ventilation with higher PEEP can be used to prevent atelectasis; however, higher PEEP could also induce overdistention of lung tissue. This could be the reason why two previous studies of high versus low PEEP in surgery patients did not show benefit of a higher PEEP strategy [1,2]. Individualization of PEEP wherein a balance between atelectasis and overdistension is achieved may be feasible [3], and the intraoperative driving pressure (ΔP) has been suggested as a target for setting the PEEP [4,5,6].

It remains uncertain how to ‘best’ titrate PEEP to ΔP. One method is to use a decremental PEEP trial. First, a recruitment maneuver (RM) using PEEP is performed. Then, PEEP is gradually decreased to identify the level at which ΔP, after an initial decrease (indicating alveolar recruitment), again starts to increase (indicating lung overdistension). After a second RM, PEEP is set at the level that corresponds with the lowest ΔP.

A potential cause for harm from this method is that the use of RMs may result in hemodynamic instability—the increased intrathoracic pressure may lead to an increase of right ventricular afterload, decreased venous return and decreased contractility of the left and right ventricle [7]. Indeed, previous studies showed that intraoperative ventilation with higher PEEP and RMs increases the risk of intraoperative hypotension [1,2,8].

Our research group is currently conducting an international multicenter randomized clinical trial (‘DESIGNATION’) using this method for titration of PEEP described above [9]. As of mid-2022, the DESIGNATION study was halfway, and all centers were actively recruiting patients. It is, however, important to monitor how often hypotension occurs in this study and how well PEEP is set according to a titration. In preparation of the Second Data Safety Monitoring Board meeting, we tested the hypothesis that the trial’s ‘individualized high PEEP strategy’ is safe and feasible.

## 2. Materials and Methods

### 2.1. Design

DESIGNATION is an investigator-initiated, international, multicenter, double-blinded randomized clinical trial that currently enrolls patients scheduled for open abdominal surgery and at increased risk for PPC in 30 centers in Europe. The study protocol and its amendments have been approved by the institutional review board of each participating hospital, and all patients are asked for written informed consent before surgery. DESIGNATION is registered at Clinicaltrials.gov (study identifier NCT03884543, accessed on 5 June 2023), and the study protocol has been prepublished [9]. In short, DESIGNATION compares an ‘individualized high PEEP’ ventilation strategy with a ‘standard low PEEP’ ventilation strategy during general anesthesia for surgery with respect to occurrence of postoperative pulmonary complications (PPC) within the first five postoperative days. Patients in the ‘individualized high PEEP group’ receive an intraoperative ventilation strategy with higher PEEP that is set according to the results of a decremental PEEP trial. The ‘individualized high PEEP’ strategy also uses RMs, at start of intraoperative ventilation, after any accidental disconnection from the ventilator, and at the end of anesthesia immediately before tracheal extubation. Patients in the ‘standard low PEEP group’, receive an intraoperative ventilation strategy with PEEP fixed at 5 cm H_2_O. The ‘standard low PEEP’ strategy does not allow standard use of RMs. Changes in PEEP, and the use of (additional) RMs, are allowed in both arms of the study, but only if strict criteria are met.

In this planned interim analysis, we explicitly did not analyze any data related to the primary endpoint of DESIGNATION, i.e., occurrence of PPC in the first five postoperative days. Here we focused on endpoints related to safety and feasibility of the intervention. The database was assessed only for these data, and we strictly adhered to a statistical interim analysis plan that was finalized before cleaning the DESIGNATION database halfway through the study. After cleaning, we exported the necessary data and subsequently locked the interim dataset.

### 2.2. Inclusion and Exclusion Criteria

Patients are eligible for participation in the DESIGNATION study if: (1) aged 18 years or older; (2) scheduled for open abdominal surgery; and (3) having an increased risk for developing PPC, using an Assess Respiratory Risk in Surgical Patients in Catalonia (ARISCAT) score of 26 points as a cutoff [10]. Patients are excluded if planned for combined abdominal and intrathoracic surgery, or surgery in a lateral or the prone position. Patients with a body mass index >40, severe cardiac disease (New York Heart Association (NYHA) III or IV, acute coronary syndrome, persistent ventricular tachyarrhythmia’s), or a reported pregnancy. Patients included in another interventional study, patients that previously participated in the DESIGNATION study, and patients who did not give informed consent were also excluded.

For this interim analysis, we excluded patients with incomplete data for the safety or feasibility endpoints, i.e., those patients who were enrolled just before closing the database.

### 2.3. Data Collected

Data collected in the DESIGNATION study included baseline characteristics, duration of surgery; details on the anesthetic procedures including type of anesthesia, type of epidural and duration of anesthesia; vital and ventilation variables including mean arterial pressure (MAP), oxygen saturation (SpO_2_), tidal volume (V_T_), set PEEP, peak pressure (Ppeak), plateau pressure (Pplat), fraction of inspired oxygen (FiO_2_), respiratory rate (RR) and end–tidal carbon dioxide (etCO_2_); whether RMs were performed; and whether intraoperative complications occurred within the preceding hour (e.g., hypotension, need for vasopressors, cardiac arrhythmia).

The vital and ventilation variables and the intraoperative complications are collected hourly and at three fixed time points, i.e., after anesthesia induction, after the decremental PEEP titration, and at the end of anesthesia before extubation. During the decremental PEEP titration, the attending anesthesiologist determines which PEEP results in the lowest ΔP. For this, they construct ΔP–PEEP graphs that are saved in the electronic database of the study.

### 2.4. Interventions

**Figure 1:**
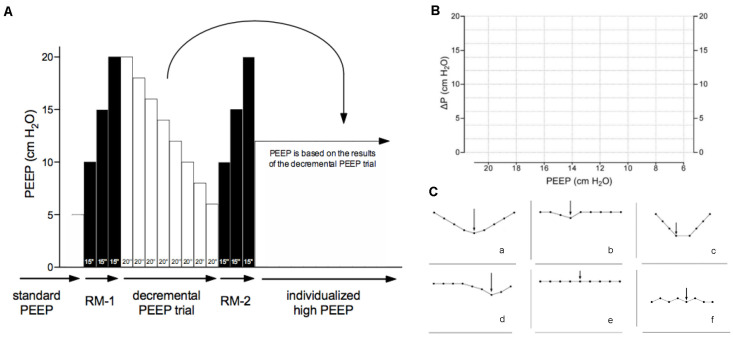
(**A**) Patients allocated to the ‘individualized high PEEP’ group receive a first recruitment maneuver (RM) followed by a decremental PEEP titration and a second RM. The RMs are performed with the ventilator in a volume-controlled ventilation modus and with the respiratory rate set at 15 breaths per minute. An inspiratory pause is manually set at 30%. Every 15 s, PEEP is incrementally increased from 5 cm H_2_O with steps of 5 cm H_2_O up to 20 cm H_2_O. The decremental PEEP titration starts at the end of the first RM at PEEP 20 cm H_2_O, with ventilator in a volume-controlled ventilation modus, and with the respiratory rate set at 15 breaths per minute. In steps of 20 s, PEEP is stepwise decreased steps of 2 cm H_2_O for 20 s until a minimum level of 6 cm H_2_O is reached. At the end of each step, the resulting ΔP is calculated by subtracting PEEP from the plateau pressure. A second RM follows the decremental PEEP titration, after which PEEP is set and kept at the highest level where ΔP was lowest. (**B**) A bedside ‘ΔP–PEEP’ graph is to be constructed by plotting the ΔP against PEEP using the empty chart. (**C**) From this ‘ΔP–PEEP’ graph, we can visually observe a nadir in ΔP, and identify the highest level of PEEP with the lowest ΔP (represented in subpanels (**a**–**d**) with the arrow). If no nadir in ΔP is present in the ΔP–PEEP graph (represented in subpanels (**e**,**f**)), we use a PEEP level of 12 cm H_2_O.

Patients allocated to the ‘individualized high PEEP’ group should receive an intraoperative ventilation strategy with high PEEP, set according to a decremental PEEP titration that is performed shortly after intubation and start of ventilation (Figure 1), with RMs at start of ventilation and within the last hour before tracheal extubation at the end of surgery. In case of any disconnection from the ventilator–patient circuit, RMs are repeated. Of note, RMs are only performed in hemodynamically stable patients, as judged by the attending anesthesiologist. The aim of the PEEP titration is to maximize alveolar recruitment while minimizing lung overdistension. In patients where the PEEP titration does not show a nadir, PEEP was set at 12 cm H_2_O as in a previous study of high versus low PEEP during intraoperative ventilation [1].

Patients allocated to the ‘standard low PEEP’ group should receive intraoperative ventilation with PEEP fixed at 5 cm H_2_O. A decremental PEEP titration is not performed in these patients, and RMs are not standardly used. For both groups, a V_T_ of 8 mL/kg predicted bodyweight is used.

### 2.5. Rescue Therapies

If any desaturation occurred, i.e., in the absence of airway problems, severe hemodynamic impairment, or a ventilator defect, a predefined rescue strategy was allowed. For instance, desaturation in patients from the individualized high PEEP group may still suggest overdistension, therefore PEEP could be decreased stepwise, and FiO_2_ eventually increased (Table 1A). In patients from the standard low PEEP group, the FiO_2_ can be increased first, eventually followed by PEEP increase and ultimately a RM.

### 2.6. Predefined and Allowed Protocol Deviations

The ventilation settings, including PEEP, can be changed at any time in case of any safety concern or in the unlikely event that PEEP interferes with the surgical procedure (Table 1B).

### 2.7. Definitions

The study protocol of DESIGNATION mandated the collection of any episode of intraoperative hypotension, defined as a reduction in mean arterial pressure (MAP) of more than 20% from the baseline measured before the induction of anesthesia, lasting for a duration longer than 3 min [9]. Excessive use of vasopressors is defined as use of any vasoactive agents, either as a bolus or continuous infusion, in quantities exceeding what was deemed necessary to counteract the vasodilating effects of the employed anesthetics, as assessed by the attending anesthesiologist. Cardiac arrhythmia is predefined as any new arrhythmias needing intervention.

ΔP was calculated as follows:

ΔP = Pplat − PEEP(1)

V_T_ was corrected for predicted bodyweight (PBW) using the following equations:

V_T_ = absolute V_T_/PBW [kg] (2)

PBW = 50.0 + 0.91 × (height [cm] − 152.4) (in males)(3a)

PBW = 45.5 + 0.91 × (height [cm] − 152.4) (in females).(3b)

The respiratory compliance (C_RS_) was calculated as follows:

C_RS_ = absolute V_T_/ΔP (4)

A patient was classified as overall study-compliant if all of the following criteria are met: (i) correct initial PEEP selection according to the study arm; (ii) correct PEEP settings over the first four hours of general anesthesia; and for patients allocated to the individualized high PEEP group: (iii) the final RM was performed, holding in mind that there could be deviations that were allowed.

The decremental PEEP titration was defined as performed when a ΔP–PEEP graph was submitted, or uploaded to the electronic database. A nadir in the ΔP during the decremental PEEP titration is defined as a drop in ΔP > 2 cm H_2_O, visualized in the ΔP–PEEP graphs.

### 2.8. Endpoints

One safety endpoint of this interim analysis was the occurrence of intraoperative hypotension. Other safety endpoints were the use of vasopressors exceeding that of what is expected to compensate for the hemodynamic effects of anesthesia and the incidence of arrhythmias. One feasibility endpoint was whether a decremental PEEP titration was performed. Other feasibility endpoints were whether there was a nadir in the ΔP, whether PEEP was set correctly, whether RMs were used according to the study protocol, and whether the RMs and decremental PEEP titration resulted in a different PEEP and ΔP.

### 2.9. Sample Size

We did not perform a formal sample size calculation for this planned interim analysis. The sample size was based on the number of enrolled patients with sufficient data for this analysis, halfway through the study.

### 2.10. Analysis Plan

Continuous variables were presented as medians with interquartile ranges and categorical variables as numbers and percentages.

Safety endpoints were reported in numbers and percentages. Safety endpoints were compared between groups using a χ^2^ test for hypothesis testing including computation of risk ratio and absolute risk difference, with 95% confidence intervals (CIs). Feasibility endpoints are reported in numbers and percentages. Feasibility endpoints were compared between groups using a Student’s *t* or Mann–Whitney *U* tests as appropriate. We reported the difference in PEEP settings, i.e., between set PEEP and the PEEP that should have been used according to study protocol. We also constructed cumulative distribution plots to show the distributions of PEEP and ΔP at the nadir of ΔP in the decremental PEEP titration, and hourly thereafter, the set PEEP and ΔP. Ventilator parameters were plotted over time, *p* values were computed for differences between groups and for differences with an interaction between group and time.

A *p* value of 0.05 was considered significant, and 95% confidence intervals are presented to express statistical uncertainty. All analyses were performed using ‘R: A Language and Environment for Statistical Computing’, version 4.1.0 (Vienna, Austria).

## 3. Results

### 3.1. Patients

**Figure 2:**
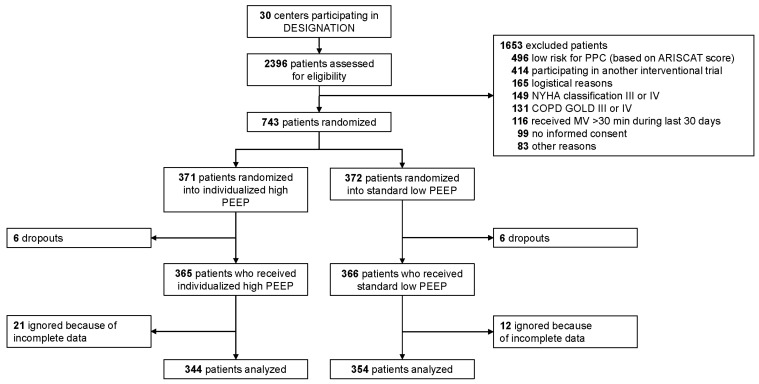
CONSORT flow diagram.

Between September 2018 and July 2022, 2396 patients were screened in 30 centers (Figure 2). Of them, 1653 patients were excluded, mainly because of a low risk for PPC or participation in another interventional study during general anesthesia. The study was stopped in 12 patients because the surgical approach was changed from open to closed after randomization. An additional 33 patients were excluded because of incomplete data for the safety and feasibility endpoints at the moment the interim database was closed. Of the remaining 698 patients, the baseline characteristics are presented in Table 2. Patients had a median age higher than 65 years, and were often obese. The majority of patients were at intermediate risk for PPC, and were classified as ASA 2.

### 3.2. Safety

Hypotension occurred more often in the individualized high PEEP group compared to in the standard low PEEP group (54.7 vs. 44.1%; risk ratio, 1.24 [95%–CI 1.07 to 1.44]; *p* < 0.01) (Table 3), and patients in the individualized high PEEP group were administered vasopressors more often (31.9 vs. 20.2%; risk ratio, 1.58 [95%–CI 1.22 to 2.04]; *p* < 0.01). The incidence of cardiac arrhythmias was similar for both groups. There was no difference in occurrence of hypotension (54.0 vs. 56.6%, *p* = 0.747) and need for vasopressors (33.7 vs. 20.7%, *p* = 0.101) in patients with a nadir in ΔP versus patients where no nadir ΔP could be identified.

### 3.3. Feasibility

The study protocol was correctly followed in 285 out of 344 patients (82.8%) in the individualized high PEEP group and in 345 out of 354 patients (97.5%) in the standard low PEEP group (Table 4). In most patients, the decremental PEEP titration identified a nadir in the ΔP, and PEEP was set accordingly in nearly all patients. The ΔP–PEEP curve did not show a nadir in 53 out of 343 (15.5%) patients. Those patients continued with 12 cm H_2_O. The most frequent protocol violation was failure to apply the RM just before extubation.

### 3.4. Ventilator Management and Ventilation Parameters

**Figure 3:**
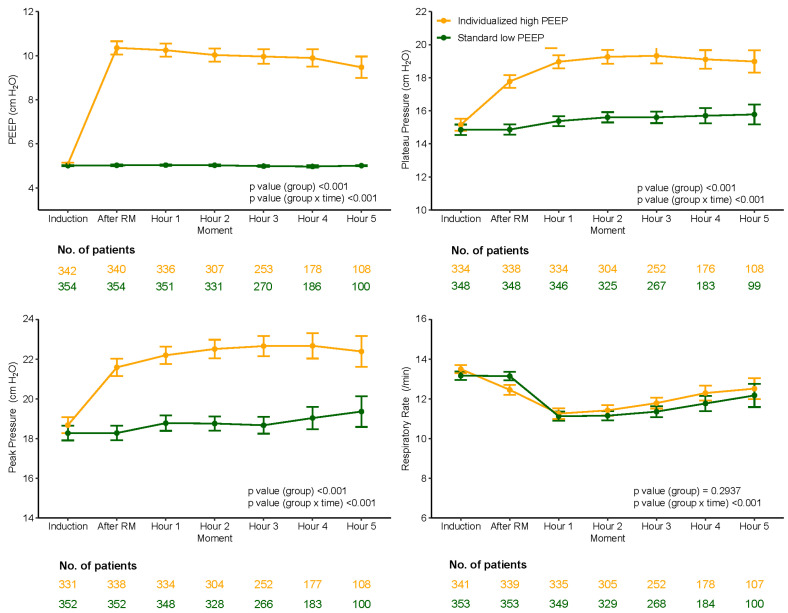
PEEP, Plateau pressure, Peak pressure, Respiratory Rate over time according to groups.

**Figure 4:**
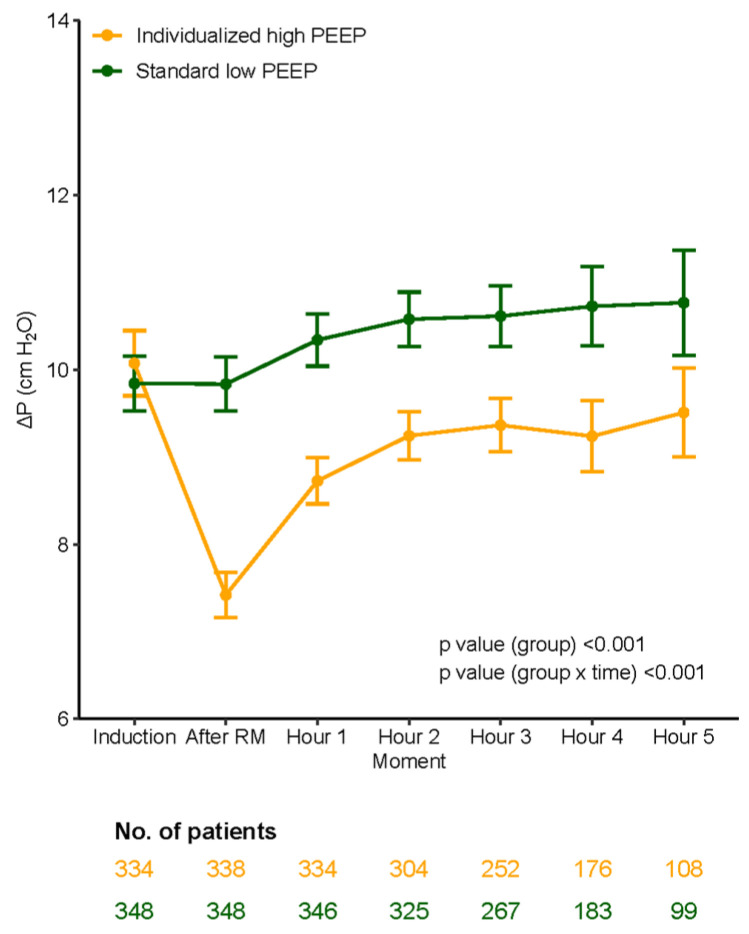
ΔP over time according to groups. ΔP, Driving pressure.

**Figure 5:**
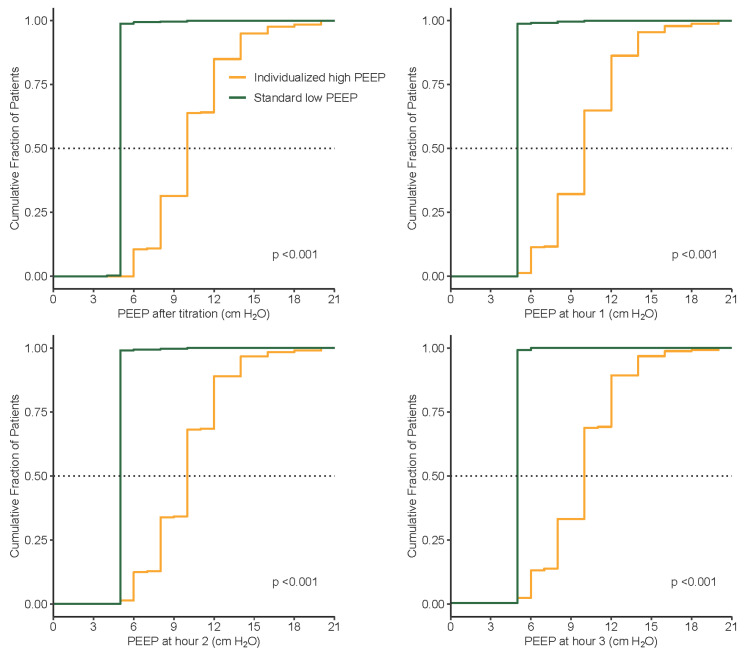
Cumulative distribution of PEEP at different time points during surgery between groups.

**Figure 6:**
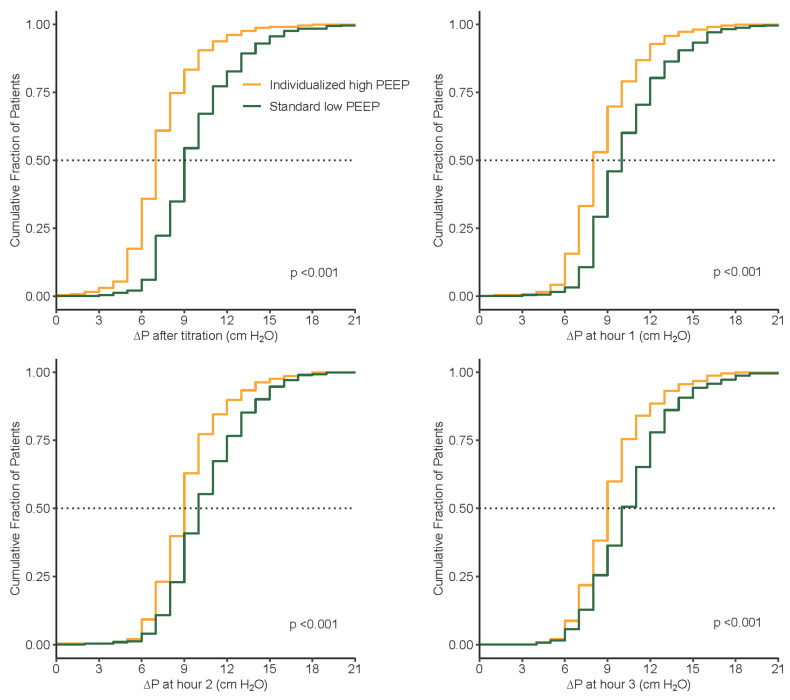
Cumulative distribution of ΔP at different time points during surgery between groups. ΔP, Driving pressure.

Compared to the standard low PEEP group, median PEEP was significantly higher and median ΔP was significantly lower in the individualized high PEEP group (Table 5, Figure 3, Figure 4, Figure 5 and Figure 6). These differences remained till the end of ventilation. Pplat, Ppeak, and C_RS_ were higher, while RR and etCO_2_, were lower in the individualized high PEEP group (Table 5).

## 4. Discussion

The findings of this interim analysis for safety and feasibility of an ongoing randomized clinical trial comparing an ‘individualized high PEEP’ ventilation strategy with a ‘standard low PEEP’ ventilation strategy in patients undergoing intraoperative ventilation during general anesthesia for scheduled open abdominal surgery can be summarized as follows: (1) hypotension occurs more often and vasopressors are more frequently used in the ‘individualized high PEEP’ group; (2) the incidence of cardiac arrhythmias is not different; (3) investigators are fairly compliant with the study protocol, wherein missing the final RM before extubation is the most frequent protocol violation; and (4) the compared strategies result in clear contrast in set PEEP.

This analysis has several strengths. We performed this analysis halfway through the study, meaning that we have a fairly large number of patients available for comparisons. The number of missing data was low, both for the analysis of the safety and feasibility endpoints. We strictly followed a predefined analysis plan that allowed us to assess safety and feasibility, and we used clear definitions as to how these endpoints were collected and measured, enhancing the validity and the reliability of the analysis.

One salient finding of this analysis is that hypotension occurs often in patients undergoing general anesthesia in open abdominal surgery. Several intravenous drugs used for general anesthesia can cause hypotension, by inducing relaxation of arterial smooth muscle cells in the peripheral vasculature [11,12]. Epidural anesthesia, which is often applied in open abdominal surgery, is another contributing factor leading to hemodynamic impairment in these patients. Drugs used for epidural anesthesia can also produce peripheral vasodilation, and promote peripheral blood pooling [13]. Of note, neither intravenous administration of drugs for general anesthesia, nor the use of epidural anesthesia differed between the two study groups. Therefore, the increased rate of hypotension found in the ‘individualized high PEEP’ group is most likely the result of using higher PEEP and RMs. Both increase intrathoracic pressure, which could impair cardiac filling and output. The increased incidence of hypotension with use of higher PEEP and RM is in line with previous clinical studies testing the efficacy of comparable strategies [1,2]. Associations have been found between intraoperative hypotension and harm, including postoperative organ dysfunction [14], such as myocardial injury [15,16,17] and postoperative renal injury [17,18], and 30-day mortality [19,20]. The finding that vasoactive drugs were used more frequently in patients receiving the ‘individualized high PEEP’ strategy confirmed results of previous reports [1,2]. Higher use of vasoactive drugs could also be harmful, as its use has been associated with an increased risk of anastomotic leakage [21,22].

Taken together, the current available evidence suggests that there might be harm from using high PEEP and RMs during intraoperative ventilation. The imminent results of the ongoing DESIGNATION study will show whether the potential benefit of high PEEP and RMs, i.e., a reduction of PPC, outweighs its potentially harmful effects. In addition, future studies should further explore the relationship between various definitions, relative or absolute, and characteristics of hypotension, such as the depth and duration, induced by high PEEP during intraoperative ventilation.

The overall adherence to the study protocol is high, especially when considering that the correct selection of a high PEEP according to a nadir in ΔP during the decremental PEEP titration could be sensitive to errors and mistakes. Nearly every allocated patient underwent PEEP titration, and nine out of ten patients received the correct level of PEEP thereafter. RMs before extubation, however, were often missed, albeit that this happened less often than in one previous study that tested the efficacy of a comparable intervention [1]. We consider this rate acceptable, also because, at the end of anesthesia, patients may have already started breathing spontaneously, which makes the proper performance of a RM nearly impossible. As expected, the investigators were very compliant in using the fixed level of PEEP in patients allocated to the low PEEP group. This level of PEEP is reported as the most often used level during intraoperative ventilation [23].

The decremental PEEP titration identified a nadir in the ΔP in eight out of ten patients, but the associated ΔP was lower than in other clinical studies in which PEEP was titrated to the lowest ΔP [8,24,25]. The difference in ΔP may be seen as small, but we would like to stress that the aim of this study is not to achieve a maximum difference in ΔP, but in PEEP wherein we target the ‘best’ high PEEP, i.e., the PEEP level at which the ΔP is lowest.

The rationale for conducting this interim analysis focusing on safety and feasibility is twofold. The DSMB requested a safety analysis due to concerns raised by previous studies indicating potential harm, specifically hypotension, associated with the intervention [1,2,8]. We have also chosen to present the feasibility findings in this manuscript. This decision was motivated by the understanding that readers, especially those considering new studies in different patient groups, need to be informed not only about the feasibility of the intervention but also about the associated risks. The patient enrollment for DESIGNATION faced significant challenges due to the COVID-19 pandemic. Elective surgeries were halted and non-COVID-19-related research was deprioritized. Therefore, this interim analysis was performed a mere 4 years after the initiation of the study.

This analysis has limitations. The method of capturing hypotension in DESIGNATION is limited. Further investigations are necessary to comprehensively examine the impacts of the tested intervention on the frequency and intensity of hypotension, with careful consideration given to the exact blood pressure levels and the specific duration of hypotensive episodes. Second, local investigators could not be kept blind for the two strategies compared, which potentially introduced observer bias; however, the investigators performing this analysis were kept blind for the randomization. Third, we did not correct for possible confounding by the intraoperative fluid management, as we did not use data concerning intraoperative fluid strategies. Fourth, in this interim analysis, we did not account for a possible center effect on the safety endpoints. Fifth, there exists a potential for underreporting of safety endpoints in the way we collected the data. Hypotension, in certain instances, might have been averted or addressed through the administration of a vasoactive drug, either as a bolus or continuous infusion. The determination of whether such intervention exceeded ‘normal’ use, i.e., quantities exceeding what was deemed necessary to counteract the vasodilating effects of the employed anesthetics, was left to the discretion of the attending anesthesiologist. This issue could have, to some extent, been mitigated by establishing a predefined threshold for excessive vasopressor usage.

## 5. Conclusions

In patients undergoing general anesthesia for open abdominal surgery, an individualized high PEEP ventilation strategy titrated to the lowest ΔP is associated with hypotension and increased use of vasopressors. The steps in the study protocol for proper setting of PEEP are feasible. The studied intervention results in clear contrast in PEEP. The DESIGNATION trial is ongoing and should finish enrollment in late 2023.

## Figures and Tables

**Table 1:** Predefined deviations from study protocol.

**(A) Rescue strategy for desaturation by group**					
**Individualized high PEEP group**			**Standard low PEEP group**		
**Step**	**PEEP**	**FiO_2_**	**Step**	**PEEP**	**FiO_2_**
1	20	0.4	1	5	0.4
2	18	0.4	2	5	0.5
3	16	0.4	3	5	0.6
4	14	0.4	4	5	0.7
5	12	0.4	5	5	0.8
6	12	0.5	6	6	0.8
7	12	0.6	7	RM	
8	10	0.6			
9	8	0.6			
10	6	0.6			
11	6	0.7			
12	6	0.8			
Downtitration of PEEP as rescue of desaturation. Starts at level of PEEP set after decremental PEEP trial.			Uptitration of PEEP and recruitment maneuvers (RM) as rescue of desaturation.		
**(B) Pre-approved protocol deviations**					
In both groups, the anesthesiologists were allowed to change the ventilation protocol at any time upon surgeons’ request or if there was any concern about the patient’s safety. If one of the following complications occur and are not responding to specific conventional therapy, the level of PEEP could be changed according to any of the following safety concerns that may occur: Persistent hypotension, not responding to treatment; Need for vasoactive drugs at higher dosages than acceptable; Need for massive transfusion, with more than five units of blood to maintain hematocrit >21% (or hemoglobin >7 mg/dL); Surgical complications determining life-threatening situations; Any deviations from the study protocol, other than those mentioned above, were considered protocol violations.					

**Table 2:** Baseline characteristics.

	Individualized High PEEP (N = 344)	Standard Low PEEP (N = 354)
Age, years	67.0 (57.0–73.0)	66.0 (57.0–72.8)
Female gender	171/344 (49.7)	181/354 (51.1)
BMI, kg/m^2^	25.5 (23.0–28.7)	25.4 (22.5–28.4)
ARISCAT score		
Intermediate (26–44)	282/344 (82.0)	278/354 (78.5)
High (>44)	62/344 (18.0)	76/354 (21.5)
Smoking status		
Never	147/344 (42.7)	163/353 (46.2)
Former	138/344 (40.1)	136/353 (38.5)
Current smoker	59/344 (17.2)	54/353 (15.3)
ASA physical status classification system		
1	23/344 (6.7)	25/354 (7.1)
2	179/344 (52.0)	205/354 (57.9)
3	140/344 (40.7)	118/354 (33.3)
4	2/344 (0.6)	6/354 (1.7)
NYHA classification		
I	111/344 (32.3)	100/354 (28.2)
II	23/344 (6.7)	29/354 (8.2)
III	3/344 (0.9)	1/354 (0.3)
IV	0/344 (0.0)	0/354 (0.0)
Not applicable	207/344 (60.2)	224/354 (63.3)
Functional status		
Non-dependent	324/344 (94.2)	338/354 (95.5)
Partly dependent	19/344 (5.5)	13/354 (3.7)
Totally dependent	1/344 (0.3)	3/354 (0.8)
History of active cancer	248/344 (72.1)	249/354 (70.3)
History of chronic obstructive pulmonary disease	20/344 (5.8)	21/354 (5.9)
With inhalation therapy	15/20 (75.0)	10/21 (47.6)
With systemic steroids	1/20 (5.0)	2/19 (10.5)
History of diabetes mellitus.	40/344 (11.6)	50/354 (14.1)
Dietary adjustments	3/40 (7.5)	1/50 (2.0)
With oral medication	21/40 (52.5)	22/50 (44.0)
With insulin	8/40 (20.0)	13/50 (26.0)
No medication	2/40 (5.0)	3/50 (6.0)
Combination of oral and insulin	7/40 (17.5)	10/50 (20.0)
History of coronary disease	13/344 (3.8)	10/354 (2.8)
History of persistent ventricular tachycardia’s	0/343 (0.0)	1/353 (0.3)
**Preoperative testing**		
Blood test		
Hb, mmol/L	8.1 (7.3–8.9)	8.1 (7.0–8.9)
Creatinine, µmol/L	75.0 (63.0–91.0)	72.0 (61.0–87.0)
Urea, mmol/L	6.0 (4.8–7.8)	6.3 (4.5–8.1)
White Blood Cell count, ×10^9^ cells/L	7.1 (5.9–8.8)	7.1 (5.6–9.2)
Preoperative SpO_2_ *, %	98.0 (96.0–99.0)	98.0 (97.0–99.0)
Preoperative transfusion	3/344 (0.9)	1/354 (0.3)
Abnormalities on chest imaging	12/119 (10.1)	23/131 (17.6)
**Perioperative variables**		
Duration of surgery ^¥^, minutes	229.0 (153.5–311.5)	211.5 (156.5–289.8)
Duration of anesthesia ^≠^, minutes	270.0 (192.5–362.5)	255.0 (190.3–338.0)
Type of anesthesia		
Volatile	84/344 (24.4)	83/354 (23.4)
Totally intravenous	204/344 (59.3)	220/354 (62.1)
Mixed (volatile and intravenous)	56/344 (16.3)	51/354 (14.4)
Use of Epidural	224/344 (65.1)	241/354 (68.1)
Thoracic	216/224 (96.4)	229/241 (95.0)
Lumbar	8/224 (3.6)	11/241 (4.6)

Data are presented as number/total number of patients (percentage) or median [1st quartile–3rd quartile]. * Measured by pulse oximetry. ^¥^ Defined as time between skin incision and closure of incision. ^≠^ Defined as time between intubation and extubation/or physically leaving the OR. Abbreviations: ARISCAT risk score, ‘Assess Respiratory Risk in Surgical Patients in Catalonia’ risk score; ASA, American Society of Anesthesiology; Hb, hemoglobin; NYHA, New York Heart Association; SpO_2_, oxygen saturation.

**Table 3:** Safety endpoints.

	Individualized High PEEP (N = 344)	Standard Low PEEP (N = 354)	Absolute Risk Difference (95% CI)	Relative Risk Ratio (95% CI) ^¥^	*p* Value *
Hypotension	187/342 (54.7)	156/354 (44.1)	10.6 (3.2 to 18.0)	1.2 (1.1 to 1.4)	0.006
Need for Vasopressors	109/342 (31.9)	71/351 (20.2)	11.7 (5.1 to 18.2)	1.6 (1.2 to 2.0)	0.001
Heart Arrhythmias	4/342 (1.2)	2/349 (0.6)	0.6 (−0.8 to 2.0)	2.0 (0.4 to 11.1)	0.447

Data are presented as number/total number of patients (percentage) or as stated otherwise; ^¥^ The relative risk ratios and 95% CIs were computed using the Wald likelihood ratio approximation test. * *p* value was calculated using the χ^2^ test or Fisher exact test whenever appropriate.

**Table 4:** Protocol compliance.

	Individualized High PEEP (N = 344)	Standard Low PEEP (N = 354)
Overall compliance *, patients		
no protocol violations, n/N (%)	285/344 (82.8)	345/354 (97.5)
protocol violations, n/N (%)	59/344 (17.2)	9/354 (2.5)
RM and PEEP titration, patients		
first RM, n/N (%)	344/344 (100)	NA
PEEP titration, n/N (%)	342/344 (99.4)	NA
second RM, n/N (%)	344/344 (100)	NA
RM before tracheal extubation, n/N (%)	306/344 (89.0)	NA
RM after disconnection, n/N (%)	10/344 (2.9)	NA
RM as rescue, n/N (%)	NA	2/354
Nadir in ΔP, patients, n/N (%)	290/343 (84.5)	NA
ΔP at nadir, cm H_2_O	7.0 (6.0–8.0)	NA
PEEP at nadir, cm H_2_O	10.0 (10.0–12.0)	NA
PEEP settings, patients		
PEEP set correctly, n/N (%)	309/344 (89.8)	350/354 (98.9)
PEEP, cm H_2_O	10.0 (8.0–12.0)	5.0 (5.0–5.0)
PEEP set too high, n/N (%)	2/344 (0.6)	3/354 (0.8)
0–2 cm H_2_O, n/N (%)	2/344 (0.6)	2/354 (0.6)
PEEP, cm H_2_O	15.0 (14.5–15.5)	6.0 (6.0–6.0)
2–4 cm H_2_O, n/N (%)	0/344 (0.0)	1/354 (0.3)
PEEP, cm H_2_O	NA	8.0 (8.0–8.0)
4–6 cm H_2_O, n/N (%)	0/344 (0.0)	0/354 (0.0)
PEEP, cm H_2_O	NA	NA
PEEP set too low, n/N (%)	33/344 (9.6)	1/354 (0.3)
0–2 cm H_2_O, n/N (%)	7/344 (2.0)	1/354 (0.3)
PEEP, cm H_2_O	10 (9.0–10.0)	4.0 (4.0–4.0)
2–4 cm H_2_O, n/N (%)	12/344 (3.4)	0/354 (0.0)
PEEP, cm H_2_O	8.0 (7.5–8.0)	NA
5–6 cm H_2_O, n/N (%)	8/344 (2.3)	0/354 (0.0)
PEEP, cm H_2_O	6.0 (6.0–6.5)	NA
>6 cm H_2_O, n/N (%)	6/344 (1.7)	0/354 (0.0)
PEEP, cm H_2_O	7.0 (6.0–8)	NA

Data are presented as number/total number of patients (percentage) or median [1st quartile–3rd quartile]. NA, Not applicable; RM, lung recruitment maneuver. * Overall study compliance was defined as a patient received all of the following (i) correct initial PEEP selection; (ii) correct PEEP settings over the first 4 h of general anesthesia; and for patients in the Individualized high PEEP group: (iii) final RM was performed. If any of the previous criteria was not fulfilled due to predefined protocol deviations, the patient was still classified as protocol compliant.

**Table 5:** Ventilator parameters after PEEP titration.

	Individualized High PEEP (N = 344)	Standard Low PEEP * (N = 354)	*p* Value
PEEP, cm H_2_O	10.0 (8.0–12.0)	5.0 (5.0–5.0)	<0.001
ΔP, cm H_2_O	7.0 (6.0–8.8)	9.0 (8.0–11.0)	<0.001
Pplat, cm H_2_O	17.0 (15.0–20.0)	14.0 (13.0–16.0)	<0.001
Ppeak, cm H_2_O	21.0 (19.0–24.0)	18.0 (16.0–20.0)	<0.001
V_T_, mL/kg PBW	8.0 (7.8–8.0)	8.0 (7.8–8.0)	0.398
FiO_2_	0.4 (0.4–0.4)	0.4 (0.4–0.5)	<0.001
Respiratory Rate, /min	12.0 (10.0–15.0)	14.0 (12.0–15.0)	<0.001
etCO_2_, kPa	4.3 (4.0–4.6)	4.6 (4.3–4.8)	<0.001
Crs, mL/cm H_2_O	71.7 (58.8–88.6)	55.4 (44.9–67.9)	<0.001

Data are presented as median [1st quartile–3rd quartile]. * As patients from the standard low PEEP group receive no PEEP titration, the ventilation parameters are taken at baseline. Abbreviations: Crs, Respiratory system compliance; etCO_2_, end tidal carbon dioxide; FiO_2_, fraction of inspired oxygen; PBW, predicted bodyweight; PEEP, positive end–expiratory pressure; Pplat, plateau pressure; Ppeak, peak pressure; ΔP, driving pressure; V_T_, tidal volume.

## Data Availability

Data will be made available upon approval of formal request at the study’s steering committee. The request shall be sent to Marcus Schultz, marcus.j.schultz@gmail.com.

## Appendix A. The DESIGNATION Investigators

Aarts, Fenne; Aarts, Leon P.H.J.; Akkerman, Ronald D.L.; Albersen, Juliette J.E; Almac, Emre; Aurilio, Catarina; Baldini, Gabriele; Ball, Lorenzo; Battaglini, Denise; Boom, Annemieke; Bos, Petra; Breel-Tebutt, Jenni; Broens, Suzanne J.L.; Brouwer, Tammo A.; Buhre, Wolfgang F.F.A.; Bulte, Carolina S.E.; Calis, Paul J.; Cascella, Marco; Coppolino, Francesco; Cuomo, Arthur; De Boer, Hans D.; De Korte—de Boer, Dianne; Diaz-Cambronero, Oscar; Van Dijck, Maarten; van Dijk, Frits; Dorland, Galina; Edward-Rutten, Gitara M.; Van Eijk, Lucas; Evers, Jos; Foti, Lorenzo S.; Gama de Abreu, Marcelo; Gasteiger, Lukas; Godfried, Marc B.; Goeden, Steffie; Helmerhorst, Hendrik J.F.; Hemmes, Sabrine N.T.; Hofland, Jan; Hol, Liselotte; Hollmann, Markus W.; Hoogenboom, Hester; Houweling, Bernard M.; Huhn, Ragnar; In ’t Veld, Bastiaan A.; Ivanov, Dimitri; Jaeger, Maxime; John, Aaron,; Karlas, Alain; Keunen, Kirsten; Koch, Rebecca; Koopman—van Gemert, Ankie W.M.M.; Koopmans, Josèph S.H.A.; Kortekaas, Minke C.; Kostelijk, Saskia; Van Lier, Felix; Macco, Gaston E.A.P; Mandelli, Maura; Marques Mari, Anabel; Martin Monllor, Raquel; Mazzinari, Guido; Van Meenen, David; Mennillo, Fabrizio; Mesman, Linda; Morariu, Aurora; Muskiet, Ernesto R.R.; Navarro-Diaz, Aitor; Neetens, Tom; Nienhaus, Johannes; Nijbroek, Sunny G.L.H.; Pace, Maria Caterina.; Passavanti, Maria Beatrice; Pelosi, Paolo; Pembele, Rene; Piazolla, Mario; Potters, Jan-Willem; Purtscheller, Thomas; Rad, Mandana; Robba, Chiara; Romagnoli, Stefano; Roth, Sebastian; Salvino, Anica; Sansone, Pasquale; Scharffenberg, Martin; Schiavoni, Lorenzo; Schultz, Marcus J.; Serpa Neto, Ary; Servaas, Sjoerd; Slotema, Thomas; Soetens, Richard H.A; Spraider, Patrick; Stamkot, André; Stolker, Robert Jan; Stroda, Alexandra; Struys, Michel M.R.F.; Swenne, Marlou; Ten Hoope, Werner; Tenge, Theresa; Ter Heerdt, Anne; Thiel, Bram; Ubben, Johannes F.H; van Os, Ben; Vermeulen, Tom; Villa, Gianluca; Visser, Ralinka; Vos, Jaap Jan; Vughts, Manon; Wittenstein, Jakob; Van Wonderen, Stefan; Wrigge, Hermann; Zeillemaker-Hoekstra, Miriam; Van der Zwan, Tim; Zwijsen, Johannes H.M.J.
